# Patent value prediction in biomedical textiles: A method based on a fusion of machine learning models

**DOI:** 10.1371/journal.pone.0322182

**Published:** 2025-04-24

**Authors:** Yifan He, Kehui Deng, Jiawei Han

**Affiliations:** Department of Humanities, Donghua University, Shanghai, China; Scuola Superiore Sant'Anna, ITALY

## Abstract

Patent value prediction is essential for technology innovation management. This study aims to enhance technology innovation management in the field of biomedical textiles by processing complex biomedical patent information to improve the accuracy of predicting patent values. A patent value grading prediction method based on a fusion of machine learning models is proposed, utilizing 113,428 biomedical textile patents as the research sample. The method combines BERT (Bidirectional Encoder Representations from Transformers) and a stacking strategy to classify and predict the value class of biomedical textile patents using both textual information and structured patent features. We implemented this method for patent value prediction in biomedical textiles, leading to the development of BioTexVal—the first dedicated patent value prediction model for this domain. BioTexVal’s innovation lies in employing a stacking strategy that integrates multiple machine learning models to enhance predictive accuracy while leveraging unstructured data during training. Results have shown that this approach significantly outperforms previous predictive methods. Validated on 113,428 biomedical textile patents spanning from 2003 to 2023, BioTexVal achieved an accuracy of 88.38%. This study uses average annual forward citations as an indicator for distinguishing patent value grades. The method may require adjustments based on data characteristics when applied to other research fields to ensure its effectiveness.

## Introduction

Patent value prediction is essential for sustainable technology innovation and investment management [1–3]. By accurately predicting patent value, stakeholders can make informed decisions that support sustainable practices in biomedical textiles. As a key indicator reflecting technological innovation and development trends, Patent data provides a rich data source for patent value prediction [4].

In areas of patent value prediction, existing methods fail to effectively use unstructured information from patent texts. In past research, curve-fitting techniques [5,6] and stochastic models [7–10] have been widely used as scientific methods in the study of patent value. However, these methods often rely on predetermined functional forms or probability distribution assumptions and are limited in their effectiveness when dealing with sparse data and processing patent text information [11]. In contrast, machine learning models can learn complex patterns from data without needing prior assumptions, effectively improving the model’s ability to predict patent value in sparse data situations [12,13]. In particular, natural language processing (NLP) techniques in machine learning can utilize the unstructured text data in patent information, conduct an in-depth analysis of the textual content, and enhance the accuracy of patent value prediction [4,11,14,15].

The key to integrating unstructured data is to convert the text into a machine-readable format, allowing the model to process and analyze it. Traditional text representation methods, such as bag-of-words models and Term Frequency-Inverse Document Frequency (TF-IDF), mainly rely on word frequency statistics and do not take contextual relationships into account, and thus are ineffective in recognizing long sequences of patent text information [16,17]. As deep learning-based word embedding techniques, Word to Vector (Word2Vec) and Document to Vector (Doc2Vec) capture part of the contextual information through a fixed-length window. However, the word vectors they generate are still static, which limits their ability to handle polysemous words and understand context [4,18,19]. For example, in static word vector models, the word “fiber” would have the same representation whether it refers to suture fibers or fibers in medical protective fabrics. These word representations are effective in topic evolution analysis based on Latent Dirichlet Allocation (LDA) and Dynamic Topic Modeling (DTM), among others, because patent topic analysis relies on studying topic words. However, an overall semantic representation of individual text passages is needed to predict patent value. Therefore, exploring NLP techniques that may be suitable for assessing patent value is essential.

The main challenge now is to integrate different types of information from patent texts to improve prediction accuracy. Given the research background, we propose a patent value grading prediction method based on a fusion of machine learning models. By applying the pre-trained language model Bidirectional Encoder Representations from Transformers (BERT), we can capture the detailed context and sequential features of patent texts. In many studies, BERT has been proven to possess the capability of deeply understanding the bidirectional context of the text [20–23]. Moreover, to further enhance the accuracy and robustness of the model in predicting patent value grades, we adopted a stacking strategy. The stacking model combines the predictions of multiple base models, leveraging their strengths to complement each other across different data patterns, thereby reducing the bias and errors of individual models. This strategy allows us to converge the predictive capabilities of multiple models by using the outputs of these models as new features input into a meta-model for making the final prediction [24,25]. In this process, we utilized patent information comprehensively, including unstructured data (such as text and titles) and structured value indicators.

Forward citation count is widely recognized as an indicator of technological impact, with numerous studies considering it a measure of an invention’s contribution to subsequent technological developments [26]. Many studies have demonstrated a significant positive correlation between forward citations and the economic value of patents [27–35]. Consequently, forward citations are frequently used as a proxy for patent value [11,18,36,37]. However, the relationship between forward citations and economic value is not always linear or direct [26]. Abrams, Akcigit, and Popadak [38] found that while forward citations generally exhibit a positive correlation with patent value, this relationship follows an inverted U-shape, suggesting that beyond a certain threshold, an excessively high citation rate may be associated with diminishing patent value. Similarly, Gambardella, Harhoff, and Verspagen [39] acknowledged that while citation-weighted measures can serve as useful indicators of patent value, they are also prone to substantial noise and inherent limitations. Hall, Jaffe, and Trajtenberg [33] further highlighted that economic value tends to be concentrated in a small subset of highly cited patents. In contrast, the majority of patents, despite accumulating citations, contribute relatively little to market value. Moreover, forward citations are influenced by various external factors, limiting their reliability as a direct measure of economic value [26]. Institutional differences across patent offices (e.g., USPTO, EPO, JPO) lead to variations in citation practices, as examiners follow distinct policies that affect citation counts [26]. Temporal and technological factors also play a role—older patents naturally accumulate more citations, while citation frequency varies across technology fields, making cross-field comparisons challenging [40–42]. Additionally, examiner heterogeneity, as noted by Cockburn, Kortum, and Stern [43], contributes to inconsistencies in citation behavior due to differences in tenure and approval times. Furthermore, Applicants strategically search for and disclose prior art, often withholding citations to optimize patent advantages [44].

Despite these limitations, forward citations remain the most widely accepted proxies for patent value [27–35]. Many prediction models rely on forward citations as a quantitative indicator to assess or rank patent value [11,36,37,45]. However, total citation counts can introduce biases, favoring older patents. To address this, the Average annual forward citations are used, as they normalize citation counts over a patent’s lifetime, allowing fairer comparisons between patents of different ages. This feature is more commonly used to rank patents rather than to predict exact future citations, with the primary goal being to assess the relative value of patents [11]. Thus, this study adopts the Average annual forward citations as the indicator for categorizing patent value. Patents are categorized into three value classes, A, B, or C, based on the average number of positive citations per year.

We applied this method to the patent value prediction of biomedical textiles, resulting in BioTexVal, a biomedical textile patent value grading prediction model. BioTexVal achieved an accuracy rate of 88.38% in identifying the value grade of biomedical textile patents from 2003 to 2023. Our goal is to improve the accuracy of patent value prediction through technological and methodological innovations, to provide new perspectives and methodologies for patent value research, and to provide practical tools and insights for technology management and decision-making.

The rest of this paper is organized as follows. The Relevant Work and Theoretical Background section presents the research background, while the Methodology section explains the research framework and methods. The Results and Discussion section presents the findings and analysis. Finally, in the Conclusion section, we present the conclusions and suggestions for future work.

## Relevant work and theoretical background

### Patent text semantic representation methods

Patent text data should be utilized to evaluate patent value [4,11,46]. In integrating patent text data into the model, the key is transforming textual information into a numerical form that machines can process, enabling the model to understand and analyze the text data. Standard text vectorization methods in patent text classification research include bag-of-words models such as TF-IDF (Term Frequency-Inverse Document Frequency) and LDA (Latent Dirichlet Allocation). For example, Wu et al. [47] used the LDA method combined with network analysis to monitor the main safety domains and technological trends in patent information, identifying technological trends to prevent various industrial system risks. Ghaffari et al. [16] employed the LDA model to analyze the share and growth rate of different technological sectors in the tire industry over two decades, 2000–2009 and 2010–2019, as well as industry and value chain-related trends and technical indicators. Shen et al. [17] used TF-IDF technology and cosine similarity analysis to identify potential patent opportunities and scientific advancements in intelligent health monitoring technology. Although TF-IDF and LDA have shown effectiveness in patent text classification and technology trend analysis, their application in patent value prediction is limited, mainly because these methods ignore the word order and contextual information in textual data.

In contrast, word embedding techniques like Word2Vec and Doc2Vec generate high-dimensional vector representations of words or documents by training deep learning models. These vectors can capture rich semantic and syntactic information, thus better reflecting the text’s true meaning. For example, Word2Vec combined with RNN (Recurrent Neural Networks) and CNN (Convolutional Neural Networks) has been used to capture sequential information and semantics in patent texts [4,19]. Additionally, techniques combining NMF (Non-negative Matrix Factorization) with Word2Vec have been applied to document clustering, preserving semantic relationships [48]. Jeon et al. [18] also transformed patent text information into vector representations using Doc2Vec technology. They combined it with the LOF (Local Outlier Factor) algorithm to propose a machine-learning method for measuring patent value on a numerical scale.

In recent years, deep learning-based pre-trained language models, particularly BERT, introduced by Google AI in 2018, have brought revolutionary changes in Natural Language Processing (NLP). The core innovation of BERT is its use of a bidirectional encoder from the Transformer architecture to pre-train deep bidirectional contextual representations, effectively capturing the complex linguistic relationships and rich semantic information in text. Devlin et al. [23] demonstrated the powerful capabilities of BERT. Furthermore, Rothe et al. [21] adapted BERT to fit biomedical corpora, significantly improving the efficiency and accuracy of biomedical text mining. Beltagy et al. [22] integrated the BERT architecture with graph neural networks, proposing an advanced automated patent classification model to support scholars’ and businesses’ technological research. Wang et al. [20] combined BERT with TRIZ (Theory of Inventive Problem Solving) and utilized patent data collected from the Derwent database to construct a technology landscape vector space model, further extending the application of BERT in the field of technology analysis.

Although BERT has strong language modeling capabilities, its complexity and high computational demands present significant challenges, especially when processing large datasets. Its deep architecture requires substantial computational resources, limiting BERT’s application in resource-constrained environments. To address this, various improved versions have been developed, such as DistilBERT and ALBERT. DistilBERT reduces model parameters through knowledge distillation, improving processing speed, but it does not perform as well as BERT in complex semantic processing [49]. On the other hand, ALBERT significantly lowers memory requirements through parameter sharing and decomposing the embedding matrix, while maintaining performance similar to BERT in certain tasks [50]. However, these lightweight models still have limitations in specific tasks [51].

Furthermore, RoBERTa and XLNet, as other improved versions of BERT, enhance performance through longer pre-training and autoregressive mechanisms. But they come with higher computational costs, limiting their applicability in resource-constrained environments [52]. Similarly, models that currently perform excellently in sentence embedding tasks—such as NV-Embed-v2 [53], bge-en-icl [54], and stella_en_1.5B_v5—also require substantial computational resources.

In contrast, BERT-base has a relatively smaller architecture (12 layers, 110 million parameters). It maintains strong text processing capabilities while requiring less computational resources. It effectively balances performance and resource consumption, making it an ideal choice in resource-limited scenarios. Therefore, this study chose BERT-base because it can provide efficient performance in patent text processing and patent value prediction while significantly reducing computational costs, making it suitable for practical applications in resource-constrained environments.

### Stacking strategy

Stacking is an ensemble technique in which a meta-learning model integrates the outputs of the base model. Stacking is often referred to as “model mixing” or simply “blending” if the final decision part is a linear model. The concept of stacking or stacked regression was introduced initially by Wolpert [55]. In this technique, the dataset is randomly divided into j equal parts. In the jth folded cross-validation, one set is used for testing and the rest for training. Using these training-testing subset pairs, the predictions of different learning models can be obtained and used as metadata to construct a meta-model. The meta-model makes the final prediction, known as the “winner-take-all” strategy.

This method of reducing bias and improving model performance has been widely applied in various fields and has been proven effective in reducing bias [25]. Initially, stacking was used to enhance the predictive accuracy of models by constructing a meta-model that combines predictions from multiple base models, particularly suitable for complex datasets. Zhang et al. [56] proposed a deep hierarchical multi-patch network for image deblurring using a stacking method. Traditional models like random forests have also been extended to deep architectures through the stacking concept, known as deep forests [57]. Zhao et al. [24] fused logistic regression, XGBoost, and BPNN models using a stacking method to build a high-accuracy enterprise financial crisis management and early warning system.

In this study, we employed eight base models chosen for their respective strengths in capturing different patterns in patent data and their ability to complement each other. Specifically, Random Forest and Extra Trees, as ensemble decision tree models, effectively reduce overfitting and are particularly suitable for handling imbalanced data. AdaBoost and CatBoost are boosting algorithms; the former excels in handling data with less noise, while the latter is optimized for categorical data, making them well-suited for patent data. XGBoost and LightGBM were selected for their outstanding performance with large-scale and sparse data. MLP, as a type of neural network, can capture complex nonlinear relationships, and KNN, being a non-parametric model, is suitable for small datasets, providing additional perspectives for our analysis.

We chose logistic regression as the meta-model because of its simplicity and good interpretability. As a linear model, logistic regression can efficiently integrate the outputs of multiple base models, avoiding overfitting issues associated with complex models. Additionally, logistic regression performs well in classification tasks since it can handle the probability outputs from the base models. Its low computational cost is advantageous, especially when stacking multiple base models, as it can quickly combine prediction results and make final classifications.

When selecting base models, we considered several common machine learning models but ultimately did not use them for various reasons. For example, SVM has long training times on large datasets and struggles to output probabilities. Naive Bayes assumes feature independence, which prevents it from capturing complex feature relationships. Single decision trees are prone to overfitting, while linear and ridge regression models have difficulty handling nonlinear data. Furthermore, although complex neural networks perform well, their high computational demands and poor generalization on small datasets make them unsuitable as base models.

Ultimately, we selected these eight base models because they can effectively handle the complexity and nonlinear relationships in patent data while balancing accuracy and computational efficiency. Choosing logistic regression as the meta-model further ensures the model’s simplicity, interpretability, and efficiency in combining multiple base model outputs. Based on the performance of the models and the consideration of computational resources, we reasonably excluded other unsuitable models, thereby constructing a robust and efficient prediction system.

### Patent value prediction based on structured patent features

As shown in Table 1, the relationship between structured patent features and patent value has been extensively explored in earlier studies, where “√” indicates a positive correlation, and “×” indicates a negative correlation. Existing methods for predicting patent value and innovativeness rely on these indicators for judgment. Most studies have found that forward citation count has a strong positive correlation with the economic value of a patent. Therefore, a patent’s forward citation count is frequently used as a quantitative proxy for its value [11,18,36,37].

**Table 1:** Research on patent value prediction based on structured patent features.

Patent value features	Correlation	Related works
IPC Classification Number Count	√	[31,58–60]
	×	[28, 29]
Claims Count	√	[27,29,39,61–63]
	×	[30]
Number of Words in the First Authority Request	√	[64,65]
	×	[66]
Forward Reference	√	[27–35]
Patent Age	√	[28,30–32,67,68]
Grant Lag	√	[31,58,69]
	×	[28]
Family Patent Count	√	[29,31,34,70]
Number of Inventors	×	[59]
Backward Reference	√	[34]
	×	[59]
Independent Inventor	√	[31,34,70]
Citations to Non-Patent Literature (NPL)	√	[71]
Patent Renewal	√	[70,72–74]

It is crucial to clarify the definition of “patent value” in this study. Generally, the economic value of an invention—whether public or private—differs from its technological impact (also known as technological value). Moreover, the value of an invention itself should be distinguished from the value of a patented invention, given that patents provide legal protection that can influence economic returns [75–77]. When considering economic value, it can be further divided into gross social value (the total benefit to producers and consumers), private value (the patent owner’s financial returns), and net social value (benefits minus any losses from displaced technologies) [26,78]. Forward citations, often used to gauge technological influence, have also been found empirically to correlate with economic value [27–35]. Researchers, therefore, frequently rely on proxies such as forward citations to estimate patent value, even though, in practice, the distinctions among various notions of value—economic versus technological—can become blurred [26]. Building on this understanding, the present study employs forward citation counts as a proxy for patent value without differentiating among these categories. In other words, the concepts—gross social value, private value, net social value, and technological value—are effectively treated as indistinguishable here. As a result, the proxy used to estimate value (forward citations) has an inherently ambiguous relationship with each definition, capturing some aspects of economic and technological value but not entirely disentangling one from the other.

In practice, employing multiple indicators together is more effective for patent valuation, reflecting its complex nature [29]. Ploskas et al. [79] implemented eight criteria for patent ranking and assessment, finding that only essential patents were identifiable with fewer than five criteria, showing a direct correlation between the number of indicators and the accuracy of patent identification. Grimaldi et al. [80] introduced a three-dimensional patent indicator combination encompassing patent strategy, commercial value, and technology for patent value assessment. Van Raan [81] suggested that a mix of various patent economic value indicators enhances the assessment of patent value, contingent on the dimensions of the patent value. Lee et al. [11] applied machine learning with a feedforward multi-layer neural network based on 21 patent indicators to delineate the intricate nonlinear relations between patent indicators and value. Chung and Sohn [4] integrated CNN and LSTM with data from abstracts, claims, and 11 other patent indicators to categorize semiconductor patent value grades. Wu et al. [47] developed an indicator system to evaluate LED patentvalue, considering technical, legal, and market conditions. Hu et al. [82] explored the potential use of machine learning models with multi-dimensional value indicator combinations to evaluate high-value patents.This study refers to the above research for the selection of input and output features. The aim is to improve the accuracy of patent value prediction by constructing a more detailed and comprehensive dataset.

## Methodology

**Fig 1:**
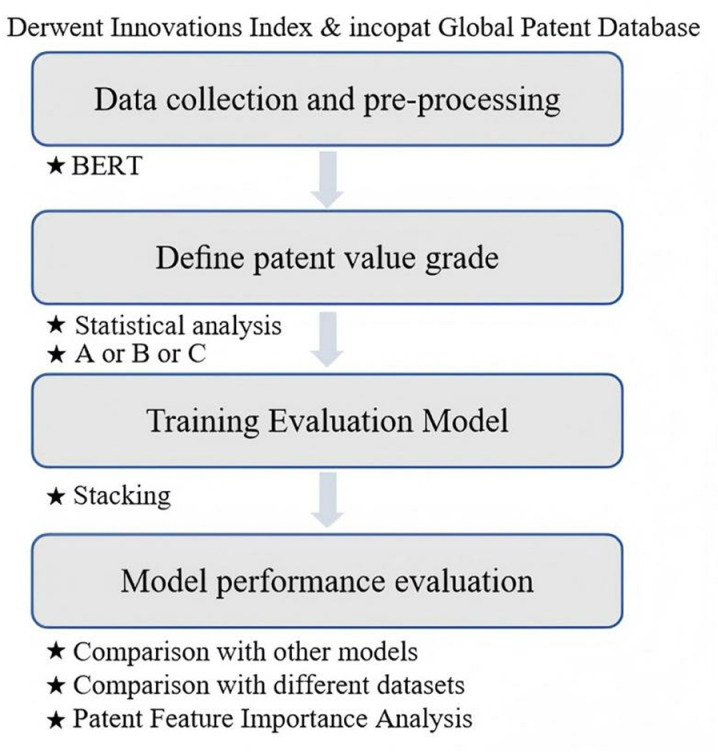
Overall process of the proposed approach.

Fig 1 illustrates the overall workflow of the proposed method, designed to enhance sustainable innovation in biomedical textiles. This method processes an extensive dataset of biomedical textile patents using advanced NLP techniques and machine learning models to predict patent value accurately. Considering the complexity involved, the proposed method is designed to be executed in four steps: data collection and preprocessing, defining patent value grades, model training, and model evaluation. By doing so, it supports efficient resource allocation and promotes sustainable practices in the industry.

### Data collection

As shown in Table 2, On November 15, 2023, we searched for biomedical textile patents in the Derwent World Patent Index (DII). The search period from January 1, 2004, to December 15, 2023, we yielded 113,428 patent records. During the search, we used the Derwent Manual Patent Code “MAN = (F04-E04)” to find relevant patents in medical textiles. In addition, the search included a series of keywords closely related to biomedical textiles, such as “antimicrobial,” “drug delivery,” “biodegradable,” “wound healing,” and “textiles.” These keywords are drawn from specialized journals and industry associations related to biomedical textiles and cover everything from material properties (e.g., “biodegradable”) to applications (e.g., “wound healing”).

**Table 2:** Biomedical textiles patent information search.

Database	Derwent World Patent Index (DII)
Time period	2004.01.01 - 2023.11.15
search queries	MAN = (F04-E04) OR TS=((‘Antimicrobial’ OR ‘Antibacterial’ OR ‘Drug delivery’ OR ‘Biocompatible’ OR ‘Biodegradable’ OR ‘Hemostatic’ OR ‘Wound healing’ OR ‘Biosensing’ OR ‘Tissue regeneration’ OR ‘Antifouling’ OR ‘Bioactive’ OR ‘Biomineralization’ OR ‘Surgical gown’ OR ‘Surgical cap’ OR ‘Face mask’ OR ‘Surgical drape’ OR ‘Surgical drapes’ OR ‘Adhesive bandage’ OR ‘Band-Aid’ OR ‘Gauze bandage’ OR ‘Protective suit’ OR ‘Protective gown’ OR ‘Protective gloves’ OR ‘Protective cap’ OR ‘Hemostatic sponge’ OR ‘Medical cotton’ OR ‘Medical gauze’ OR ‘Sterile medical gloves’ OR ‘Implant materials’ OR ‘Cardiac repair materials’ OR ‘Tissue repair materials’ OR ‘Intraocular filling materials’ OR ‘Nerve patch’ OR ‘Artificial esophagus’ OR ‘Artificial blood vessel’ OR ‘Artificial joint’ OR ‘Artificial urethra’ OR ‘Artificial valve’ OR ‘Artificial kidney’ OR ‘Artificial skull’ OR ‘Artificial heart’ OR ‘Artificial tendon’ OR ‘Artificial larynx’ OR ‘Artificial skin’ OR ‘Artificial cornea’ OR ‘Vascular Stent’ OR ‘Prostate stent’ OR ‘Biliary stent’ OR ‘Esophageal stent’) AND (‘textile’ OR ‘fiber’ OR ‘fabric’))
Result	113,428 patents

In addition, we obtained other dimensional characteristics for 97,795 patents from the INCOPAT database. During the data collection process, we obtained as much feature information as possible, ultimately collecting 205 features. In the subsequent feature selection process, some excluded features were primarily due to the following reasons:

1. Lack of effective information within the data dimension;
2. Missing rate of the data dimension exceeding 95%;
3. Redundancy between data dimensions.

For example, features such as “parent case,” “divisional case,” and “invalid applicant” only appeared in a few patents. Additionally, features like “business registration number,” “inventor address,” and “business alias” had too many categories and did not provide valuable predictive information. Based on these factors, we conducted a strict screening of all features, ultimately retaining 34 features out of the initial 205. This effectively reduced feature redundancy and noise in the model. We thoroughly examined all the feature dimensions and eliminated the invalid ones. Eventually, we constructed a dataset of 113,428 samples of biomedical textile-related patents, each with 34 input features and one output feature, where the output feature represents the Average annual forward citations. S1 Table lists these features with their respective IDs.

Although we made every effort to minimize redundancy and noise in the model, retaining 34 features still posed a potential risk of overfitting. To address this challenge, we introduced cross-validation techniques into the model. Specifically, we employed 5-fold cross-validation to ensure that the model has good generalization capabilities and can perform stably on data outside the training set, effectively preventing overfitting.

Additionally, we used model stacking strategy to further reduce the risk of overfitting. By combining the prediction results of multiple base models, model stacking can integrate the strengths of each model, reducing the bias and variance of individual models. This enhances the robustness and generalization ability of the overall model.

### Data preprocessing

**Fig 2:**
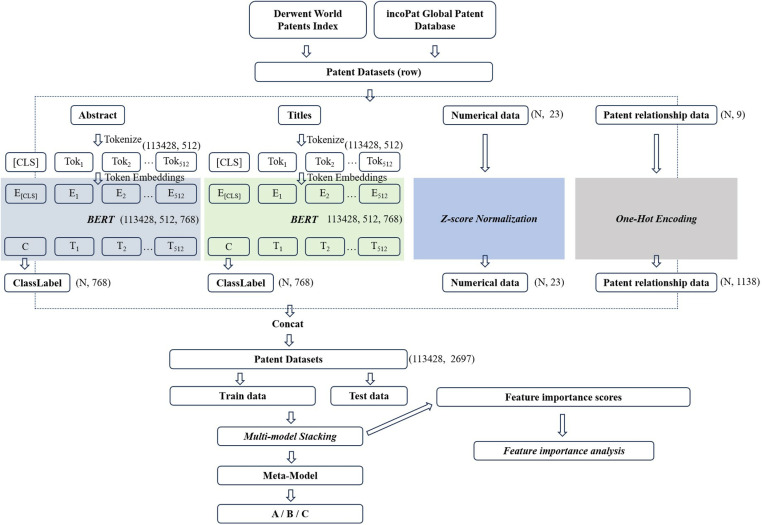
Data processing flow.

This section describes the various data preprocessing steps. As shown in Fig 2, the input data is divided into four main parts: the abstract, the title, numerical data (such as the number of claims and pages), and patent relationship data (including legal status and patent type). We have processed these four parts accordingly. Finally, these four data types were effectively integrated to construct the dataset used for training. The detailed process is described in the following section.

#### Text feature preprocessing.

S2 Table lists some examples of the collected text data. BERT is used to process the textual features of the dataset (patent abstracts and titles) to generate their semantic representations. This processing flow is detailed in Fig 2. First, the abstract and title texts are tokenized and transformed into tokens the model can understand. At the same time, the text length was normalized to meet the input requirements of the BERT model. This included removing punctuation and stop words and limiting the length to 512 tokens to ensure they did not exceed the maximum sequence length limit of the BERT model. Next, we used the `Bert-base’ model in the word embedding phase, a relatively minor version of the BERT series. Each token was mapped onto a 768-dimensional vector space. Thus, the initial embedding vector for each sequence contained a “[CLS]” vector, followed by the text token vectors. These embedding vectors were then fed into the `Bert-base’ model, which consisted of 12 layers of transformer structures. Within the model, each Transformer layer processes the input token vectors, integrating contextual information through the self-attention mechanism, thus continuously updating and optimizing the representation of each token. At the final layer of the model, “C,” which stands for “Class Label” (113428, 768), is extracted and used as input for downstream tasks.

#### Numerical feature preprocessing.

We performed z-score normalization on the 23 numerical feature dimensions in the dataset. This process converts each feature value into the number of standard deviations relative to the mean of its feature column. The calculation is as follows:


$$Z=\frac{\left(X-\mu \right)}{\sigma },$$ (1)

For a given raw data point X, the mean of the raw data is denoted μ, and the standard deviation of the raw data is σ. The standardized data point is denoted as Z. This normalization transforms the data into a distribution with a mean of 0 and a standard deviation of 1. This method effectively reduces differences between scales, handles outliers, and standardizes the data. Finally, all null and missing values were replaced with 0 to maintain the integrity and consistency of the dataset.

Missing data were replaced with zero values. Although this method is simple, it is reasonable in certain cases, especially when missing values can be interpreted as the feature not existing or being inapplicable. For example, in our dataset, missing values in certain fields often indicate that the feature does not exist in a specific patent. Therefore, replacing them with zero values is a logical choice. This approach effectively avoids imputation’s complexity while preserving the features’ actual meaning.

We compared several common normalization methods, such as min-max scaling. We ultimately selected z-score normalization because it maintains the distribution characteristics of features in high-dimensional patent data, enhancing the model’s stability and generalization ability. Z-score normalization transforms numerical features to have a mean of zero and a standard deviation of one, eliminating the effects of different feature scales. This normalization method can effectively improve the convergence speed and accuracy of many machine learning models, especially those involving gradient descent optimization algorithms, where z-score normalization can balance the influence of different features on the model.

#### Preprocessing of patent relationship data features.

We used the one-hot encoding method to process the patent relationship features in the dataset. Specifically, each unique category value within these features was converted into a series of independent binary features, allowing the original categorical information to be represented numerically in the model. This approach ensured effective conversion and encoding of the categorical features and maintained independence between features. Finally, these different feature vectors were horizontally concatenated to form a comprehensive dataset for patent analysis.

### Defining patent value grades

This section analyzed the growth in patent applications and the average annual forward citations for patents in the biomedical textiles field from 2004 to 2023. Based on these analyses, biomedical textile patents were classified into three value classes: high value (class A), medium-high value (class B), and low value (class C).

**Fig 3:**
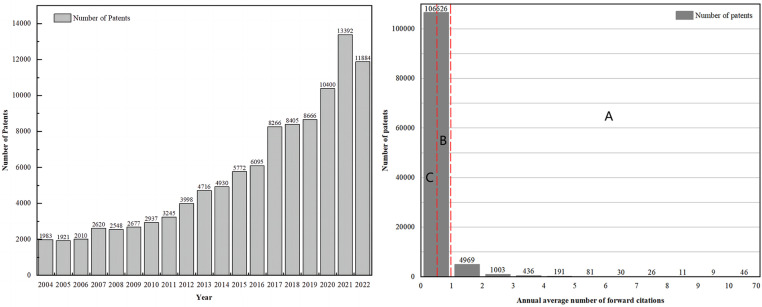
(a) Average annual number of forward citations for biomedical fabric patents; (b) Grading the average annual number of forward citations for biomedical fabric patents.

As shown in Fig 3(a), from the beginning of 2004–2023, the number of biomedical textile patent applications shows a significant growth trend, increasing at an average annual growth rate of about 11.15%. The compound annual growth rate (CAGR) during this period was 10.46%, reflecting the industry’s continuous development and technological innovation. The increasing number of patents indicates the growing investment in research and development and market demand for biomedical textile technology, making this study crucial for the sector. Fig 3(b) shows the distribution of average annual forward citations for biomedical textile patents from 2004 to 2023, demonstrating the variation in technological value between patents. Many patents have lower average annual forward citations, suggesting they may have limited market impact or are technologically more conventional. The long-tail nature of this distribution suggests that although only a few patents have received many citations, they may be critical drivers of technological change.

The study identified patent value classes by analyzing the forward citation distribution of biomedical textile patents. This feature is strongly positively correlated with the economic value of a patent, as previous studies have shown [60,83]. As a result, forward citations are often used as a quantitative feature to assess the value of patents [11,18,36,37]. This method of using average annual forward citations helps to mitigate the time bias inherent in relying solely on citation counts, as it accounts for the potential overvaluation of older patents that have had more time to accumulate citations and the potential undervaluation of newer patents with less time on the market. Despite mitigating age-related bias by normalizing citation counts over a patent’s lifetime, average annual forward citations still exhibit notable limitations, as forward citations do not necessarily correspond to a patent’s actual economic value.

Using inconsistent percentage ranges in patent value classification is intended to reflect the non-uniform distribution of patent values. Specifically, the number of forward citations a patent receives (i.e., the number of times it is cited by subsequent patents) typically follows a power-law distribution or a long-tail distribution. This means that a small number of patents are frequently cited, while the majority receive relatively few citations. This distribution pattern implies that high-value patents constitute only a minority, whereas low-value patents make up the majority.

To assess patent value, as shown in Fig. 3(b), we divided 113,428 patents into three value grades. These grades were based on the average annual forward citation distribution. Grade A represents the top 6%, Grade B covers the middle 40% (6% - 46%), and Grade C includes the remaining 54% (46% - 100%). In this scheme, grade A patents are considered high value, grade B as medium-high value, and grade C as low or no value. This classification supports the identification of high-value patents, facilitating sustainable investment decisions.

There is no universally accepted standard in academia for classifying patent value based on citation counts, leading to varying percentile cutoffs and numbers of patent categories across different studies. For instance, Lee et al. rank patents into four levels (L1–L4) using total citations [11], whereas Chung and Sohn employ average annual forward citations to divide patents into three levels (A–C) [4]. While these categorizations are informed by empirical observations, they inevitably involve subjective decisions that reflect the characteristics of the underlying data. We believe that the different choices made in previous research are primarily driven by these data considerations. Following this perspective, we determined our classification cutoffs by examining the distribution of average annual forward citations in our dataset, ensuring that the selection is both data-driven and capable of capturing meaningful distinctions in citation impact.

As illustrated in Fig. 3(b), selecting the top 1% would yield an excessively narrow set of high-value patents with limited statistical power. However, choosing the top 10% would be too broad and encompass patents whose citation impact is insufficiently distinct. Initially, we considered defining Grade A as the top 5% of the citation distribution. However, closer inspection revealed virtually no difference in citation intensity between patents in the top 5% and those in the top 6%, indicating that these two brackets share a similar level of impact. To avoid artificially splitting this high-impact group, we expanded the Grade A threshold to include the top 6%. The classification of Grades B and C follows the same rationale. This adjustment ensures that all patents with equivalent citation strength are classified together, thereby preserving coherence and accuracy in our ranking. By accurately capturing patents of truly high impact while maintaining a sufficiently large sample, this data-driven choice reinforces meaningful distinctions in the dataset and enhances model identification. In other words, setting the cutoff at 6% successfully isolates a clearly defined high-impact group without sacrificing the robustness or utility of our predictive models.

### Training evaluation model

**Fig 4:**
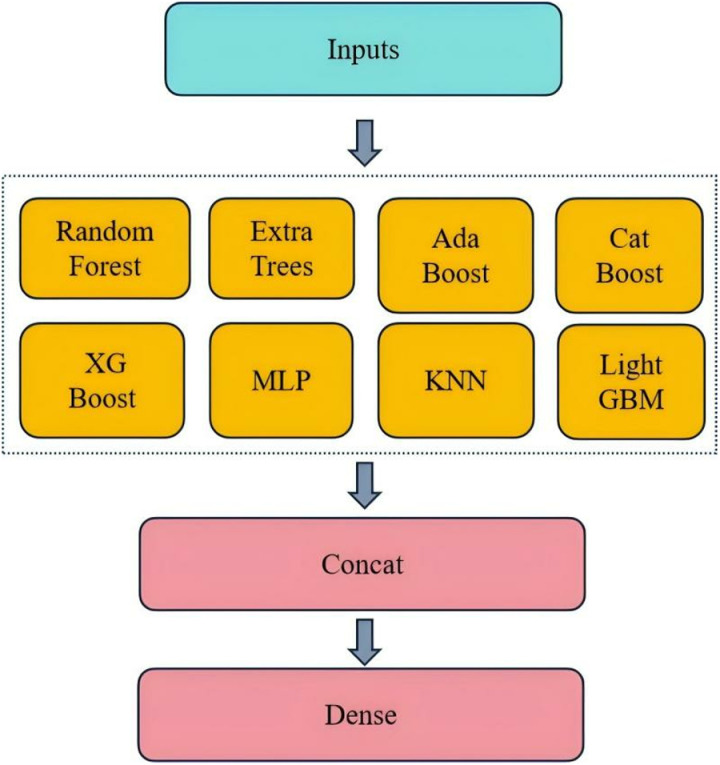
Stacking strategy.

**Fig 5:**
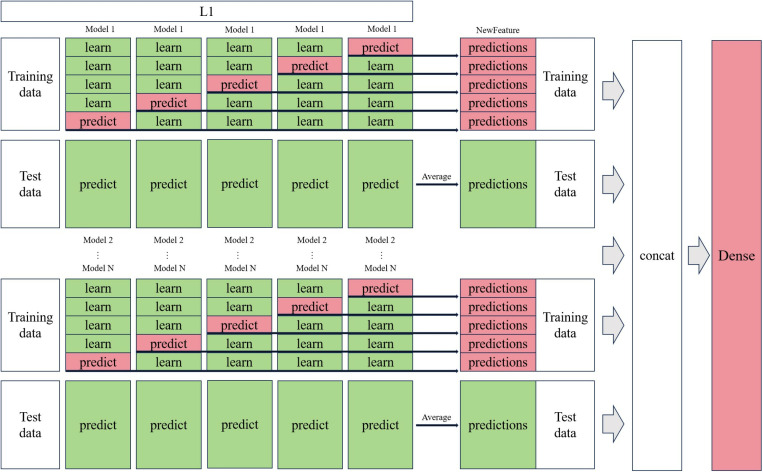
Five-fold cross validation.

The dataset was divided into 75% for training and 25% for validation (Table 3). Eight basic models, including Random Forest, Extra Tree, AdaBoost, CatBoost, XGBoost, Multi-Layer Perceptron (MLP), K-Nearest Neighbour (KNN), and Lightweight GBM, were then fused using the stacking strategy as shown in Fig 4. These models were independently trained on the training data and merged to generate predictions through the Concat layer. In addition, 5-fold cross-validation is applied in the stacking strategy to ensure that our base models fit the training dataset well and maintain accuracy and consistency when using new, unseen data. Fig 5 shows five-fold cross-validation, randomly dividing the entire training set into five equal parts. Four parts are used for training in each round, and the remaining is used for validation. This process is repeated five times, with a different part selected as the validation set and the remainder used for training. The merged feature vectors were then passed to the dense layer to further refine and integrate the knowledge from each base model.

**Table 3:** Training and validation sets.

Grade	Training set (75%)	Validation set (25%)	Total
A	5101	1701	6802
B	34393	11464	45857
C	45577	15192	60769
Total	85071	28357	113428

Finally, logistic regression was used as a meta-model to make final predictions based on the inputs from the dense layer. This meta-model is responsible for learning the best combination of predictions from each base model. Finally, our model was used to predict the patent class of the test data.

## Results and discussion

In this section, we compared the performance of different models using a dataset containing topics, abstracts, patent numerical data, and patent relationship data. In addition, we compared other models that used machine learning methods in previous studies. Finally, we analyzed the characteristics of patent value.

### Model training results and multi-model performance comparison

The models were evaluated using the following criteria: Accuracy, Precision, Macro Precision,


$$Accuracy=\frac{\sum_{i=1}^{C}TP_{i}+TN_{i}}{\sum_{i=1}^{C}TP_{i}+FP_{i}+TN_{i}+FN_{i}},$$ (2)

Recall, Macro Recall, F-1 Score, and Macro F-1 Score. Our calculations are as follows:


$$Precision_{i}=\frac{TP_{i}}{TP_{i}+FP_{i}},$$ (3)

$$\text{Macro Precision=}\frac{\sum_{i=1}^{c}\frac{TP_{i}}{TP_{i}+FP_{i}}}{c},$$ (4)

$$Recall_{i}=\frac{TP_{i}}{TP_{i}+FN_{i}},$$ (5)

$$\text{Macro Recall=}\frac{\sum_{i=1}^{c}\frac{TP_{i}}{TP_{i}+FN_{i}}}{c},$$ (6)

$$F-1score_{i}=2\times \left(\frac{Precision_{i}\times Recall_{i}}{Precision_{i}+Recall_{i}}\right),$$ (7)

$$MacroF-1score=\frac{\sum_{i=1}^{c}F-1score_{i}}{c}.$$ (8)

The categories in this study are the patent grades: A, B, or C. For example, TP_A_ represents the number of true positives for grade A, TN_A_ represents the number of true negatives for non-grade A, FN_A_ represents the number of false negatives for grade A, and FP_A_ represents the number of false positives for non-grade A. The same is true for grades B or C, and so on.

Compared to other models, the BioTexVal model, which was trained on topics, abstracts, patent numerical data, and patent relationship data, had the highest accuracy of 88.38%. Other features such as macro accuracy (84.29%), macro recall (80.49%), and macro F1 score (82.05%) also generally outperformed other models. As shown in Table 4, in BioTexVal, the precision and recall for level A are 75.62% and 61.08%, respectively. For level B, the precision and recall for each category are 82.60% and 90.73%, respectively. For level C, the precision and recall for each category are 94.66% and 89.67% respectively.

**Table 4:** Performance matrices for other machine learning models.

Model	Measure	A grade (%)	B grade (%)	C grade (%)	Macro -average (%)	Accuracy (%)
RandomForest	Precision	90.00	76.34	90.79	85.71	83.98
	Recall	1.06	88.97	89.49	59.84	
	F1-Score	2.09	82.17	90.14	58.13	
ExtraTrees	Precision	80.00	74.94	81.92	78.95	79.07
	Recall	0.47	75.66	90.44	55.52	
	F1-Score	0.94	75.30	85.97	54.07	
AdaBoost	Precision	56.33	80.52	92.16	76.34	85.36
	Recall	47.09	85.54	89.51	74.05	
	F1-Score	51.30	82.95	90.82	75.02	
K-Nearest Neighbors	Precision	36.91	68.69	79.85	61.82	73.98
	Recall	17.99	70.31	83.02	57.11	
	F1-Score	24.19	69.49	81.40	58.36	
Multi-Layer Perceptron	Precision	52.06	79.19	89.05	73.43	82.42
	Recall	62.43	78.75	87.43	76.20	
	F1-Score	56.78	78.97	88.24	74.66	
XGBoost	Precision	75.66	81.78	94.88	84.11	88.10
	Recall	57.20	91.23	89.20	79.21	
	F1-Score	65.15	86.25	91.95	81.12	
LightGBM	Precision	75.80	81.94	95.28	83.45	88.32
	Recall	59.85	91.67	88.98	80.17	
	F1-Score	66.89	86.53	92.02	81.81	
CatBoost	Precision	76.05	81.95	94.99	84.34	88.25
	Recall	57.50	91.43	89.29	79.41	
	F1-Score	65.48	86.44	92.05	81.32	
BioTexVal	Precision	75.62	82.60	94.66	84.29	88.38
	Recall	61.08	90.73	89.67	80.49	
	F1-Score	67.58	86.47	92.10	82.05	

In addition, as shown in Table 5, we tested the effectiveness of different types of patent data in the machine learning model using the XGBoost model as an example. These results indicate that machine learning models can indeed classify patents based on textual information. Although the model trained solely on textual data (i.e., titles and abstracts) achieved an accuracy of 60.02% and an F-1 score of 57.97%—lower than the model using numerical data—it still highlights the importance of textual information in patent classification. Textual data contains the core content and innovations of patents, providing valuable features for the model.When we combined textual information with structured patent indicators, the model’s accuracy significantly improved to 88.10%, with a macro F1-score of 88.04%. This further demonstrates that the integration of textual information with other features can enhance the model’s performance.

**Table 5:** Results of XGBoost evaluation (Accuracy and F-1 score).

Data Sources	Accuracy	F1-Score
(1) Title + Abstract +Numerical data + Patent relationship data	88.1	88.04
(2) Title + Abstract	60.02	57.97
(3) Numerical data + Patent relationship data	86.47	86.45
(4) Numerical data	85.1	85.04
(5) Patent relationship data	66.47	64.61

Our results show that integrating structured features and patent text information can improve the accuracy of patent value prediction in the field of biomedical textiles. Utilizing BERT-trained patent text information significantly enhances prediction accuracy. In addition, BioTexVal, using a stacking strategy, demonstrated superior performance in patent value prediction compared to individual models. These findings contribute to a deeper understanding of the theory of patent value prediction for biomedical textiles and provide a powerful analytical tool for technological innovation and management in this field. This method can support sustainable technology innovation management by more accurately identifying high-value patents, ensuring efficient use of resources.

### BioTexVal

The main objective of this study is to identify valuable patents, specifically Class A and Class B patents, while excluding low-value Class C patents. For Class A and B patents, greater emphasis is placed on Recall because these patents have higher levels of technological innovation and economic value. Minimizing false negatives allows for better identification of potential high-value patents. In contrast, for Class C patents, the focus is more on Precision to avoid misclassifying more valuable patents as low-value ones (false positives). This ensures the model accurately excludes low-value patents and enhances overall screening efficiency.

According to the results in Tables 4 and 6, the model performs exceptionally well in identifying low-value patents, achieving a precision of 94.66% for Class C patents. This indicates that the model can effectively exclude low-value patents and avoid mistakenly classifying them as high-value patents.

**Table 6:** Confusion matrix of BioTexVal.

	A (Predicted)	B (Predicted)	C (Predicted)
A (Actual)	1039	645	17
B (Actual)	312	10401	751
C (Actual)	23	1546	13623

However, the model’s performance on Class A patents is relatively weaker, with a precision of 75.62% and a recall of 61.08%. This suggests that the model has some issues with missing Class A patents. Since Class A patents represent the highest level of technological innovation and economic value, future research should focus on improving the recall rate for Class A patents to reduce the number of missed high-value patents.

For Class B patents, the model achieved a precision of 82.60% and a recall of 90.73%, demonstrating its stability in identifying moderately valuable patents. Future research can aim to optimize the recall rate for Class A patents while maintaining high predictive performance for Class B and Class C patents.

### Patent value feature analysis

**Fig 6:**
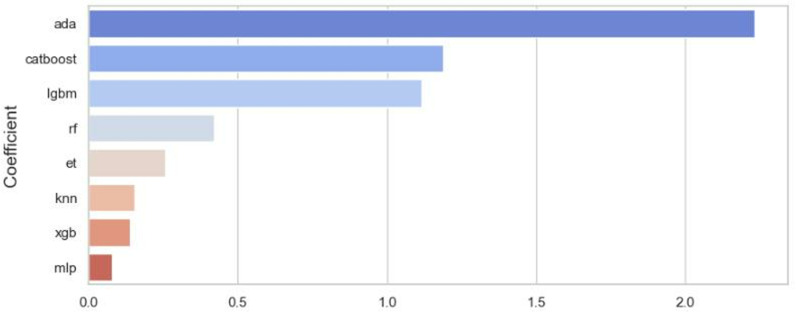
Contribution of base models to BioTexVal.

Fig 6 illustrates the impact of different base models on BioTexVal, while Table 7 presents the analysis results of feature importance for the top three base models. It is evident that certain key features consistently demonstrate significant importance in predicting the value of biomedical textile patents. The number of patent families holds high weight across all models, particularly prominent in the lgbm(LightGBM). This indicates that the number of patent families serves as a core indicator for measuring the global influence and market potential of a technology. A broad patent family not only enhances the market value of the technology but also provides more robust protection for innovations. The scope of patent protection also stands out in the CatBoost and AdaBoost models, suggesting that extensive patent protection can effectively increase the commercial value of patents, prevent competitors from entering the market, and maintain long-term competitive advantages. Compared to other technological fields, the biomedical textile sector places special emphasis on the breadth of patent protection.

**Table 7:** Feature importance ranking (Top20).

Rank	AdaBoost	CatBoost	lgbm
1	Number of Family Patents	Scope of Patent Protection	Number of Family Patents
2	MONTH	Number of Family Patents	Year
3	Scope of Patent Protection	MONTH	Number of Family Patents
4	Transfer Count	Transfer Count	Number of Cited Patent Documents
5	Number of Cited Scientific Publications	Number of Cited Scientific Publications	Technical advancement
6	Technical Stability	Inventor’s country/region_China	Page
7	Number of Cited Patent Documents	Publication country_Taiwan, China	Publication country_China
8	Abstract551	Publication country_World Intellectual Property Organization (WIPO)	Shared Value
9	Abstract522	Publication country_Eurasian Patent Organization (EAPO)	IPC Classification Number
10	Abstract519	Technical advancement	Technical Stability
11	Abstract496	Number of claims	Scope of Patent Protection
12	Abstract493	LAN_Czech	Number of claims
13	Title685	Patent Type Utility Model	Legal event_Dual Application for the Same Case
14	Abstract435	Legal event_preservation	Publication country_Japan
15	Abstract410	LAN_Chinese	LAN_Japanese
16	Abstract362	LAN_Pages	Number of Cited Scientific Publications
17	Abstract488	LAN_Finnish	LAN_nan
18	Abstract314	Number of Cited Patent Documents	Number of Derwent manual codes
19	Abstract57	DC_A11	Word Count of the First Claim
20	Abstract72	Number of Derwent manual codes	LAN_English

Additionally, the number of transfers is another important feature repeatedly identified across multiple models, especially showing high weight in AdaBoost and CatBoost. Patent transfers typically indicate high market demand and application value of the technology, reflecting the commercialization process of biomedical textile technologies. Different models focus on different features; for example, the AdaBoost model places more emphasis on time-related features such as the month and the number of patent families, highlighting its advantage in handling dynamic data related to the technology lifecycle. The CatBoost model focuses more on geographical features, such as the inventor’s country and the publication country, demonstrating its strong ability to capture regional innovation behaviors and their impact on patent value. Meanwhile, the LightGBM model emphasizes structured features like the number of patent citations, technological stability, and technological advancement, which directly reflect the depth of the technology and its market influence.

The importance of these features reveals the innovation trends in the biomedical textile field. Globalization and multinational expansion are evident trends, as indicated by the high weight of the number of patent families and the inventor’s country. This suggests that innovators need to file patents globally to enhance the market value of their technologies. Continuous technological improvement is also crucial, as the high weight of technological stability and technological advancement indicates that biomedical textile technologies rely on ongoing application and enhancement rather than single technological breakthroughs.

Compared with existing literature, some features in this study are consistent with previous research. The number of patent families and the scope of patent protection perform prominently across multiple models, highlighting their importance in assessing the global impact and market competitiveness of patents. Technological advancement and technological stability, as proprietary indicators provided by the incoPat database, show high importance in this study, reflecting the field’s need for continuous technological improvement and stable application. The significance of the number of transfers in the AdaBoost and CatBoost models reflects the close relationship between patent market circulation and value, a feature that is less discussed in existing literature.

Although semantic features are relatively secondary in all models, they enhance the prediction accuracy of the models when used as supplementary structured features.

### Error analysis

**Fig 7:**
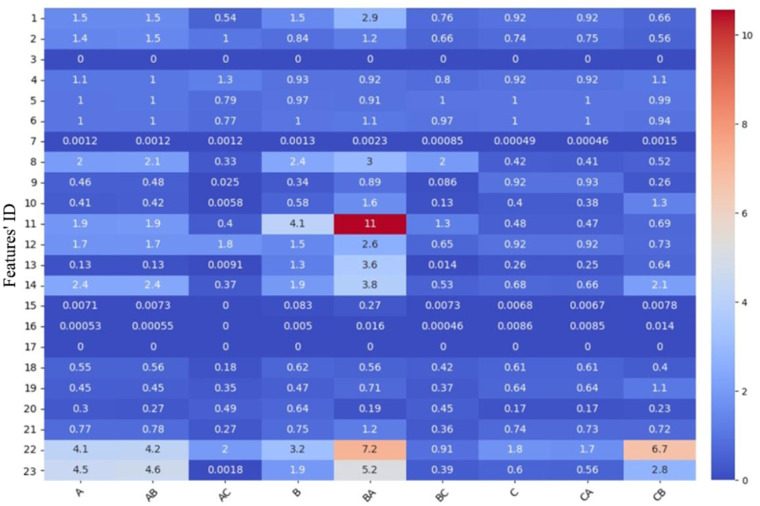
Comparison of feature variances in correct vs. incorrect sample classifications.

In feature importance analysis, we found that numerical features play a decisive role in the model’s classification of samples. Therefore, in our error analysis, we primarily focus on numerical features. After applying Z-score normalization, the variance of the data serves as an indicator of each feature’s relative volatility. By calculating the variance separately for correctly and incorrectly classified samples, we can identify the key features that influence the model’s misclassifications. Fig 7 shows the calculation results. On one hand, in incorrectly classified samples, a high variance indicates that the feature values are widely dispersed, making it difficult for the model to find a clear decision boundary. On the other hand, if a feature exhibits a significant difference in variance between correctly and incorrectly classified samples, it suggests that this feature may be a key factor contributing to the model’s misjudgments.

When class A samples are misclassified as class B (AB), the feature variances of correctly classified and misclassified samples are essentially the same, indicating that the degree of fluctuation in these feature values is similar in both cases. This situation also occurs with class C samples misclassified as class A (CA). Therefore, the volatility of the features is not the main cause of the model’s misclassification. We believe this may be because the existing features have failed to effectively distinguish the misclassified samples. It may be necessary to introduce new, more discriminative features or adopt more complex models and algorithms to improve classification performance.

Table 8 presents the features that exhibit significant differences in variance between correctly classified and misclassified samples. In cases where class A samples are misclassified as class C (AC), the model is primarily influenced by features such as IPC Classification Count (1), Claims Count (8) and Number of Words in the First Claim (11). These features display significant differences between class A and AC samples; specifically, the variance of these features is notably higher in class A samples than in AC samples, indicating that they are important indicators for distinguishing between different categories. This phenomenon also occurs when class B samples are misclassified as class C (BC). The model may be unable to accurately capture the low variance of these features in AC and BC samples, leading to classification errors.

**Table 8:** Features with significant variance discrepancies affecting classification accuracy.

Error Classification	Features
AC	IPC Classification Count (1), Claims Count (8), Number of Words in the First Claim (11), Family Patents Counts (14), Cited Patent Documents Count (22), Cited Scientific Publications Count (23)
BA/BC	IPC Classification Count (1), Claims Count (8), Number of Words in the First Claim(11), Number of Inventors (12), Number of Family Citations (13), Family Patents Counts (14), Cited Patent Documents Count (22), Cited Scientific Publications Count (23)
CB	Dependent Claims Count (9), Dependent Claims Count (10), Family Patents Counts (14), Technical Advancement (19), Cited Patent Documents Count (22), Cited Scientific Publications Count (23)

When class B samples are misclassified as class A (BA) and class C samples are misclassified as class A (CA), the increased variance in these misclassified samples indicates that the features exhibit a wider range of values compared to those in correctly classified samples. This higher variability may confuse the model, making it more difficult to establish clear decision boundaries between classes. To address this issue, additional feature engineering may be necessary. This could involve normalizing the data, removing outliers, or selecting features with lower variance to improve the model’s performance.

To further improve the model’s classification accuracy, future work may consider exploring more refined feature engineering methods from multiple perspectives, such as employing time-series models (e.g., LSTM) to offer deeper insights into patent lifecycle risks; combining dependency parsing (e.g., Stanford CoreNLP) with graph neural networks (GNN) in claim analysis to capture the topological structure of textual data for more precise assessments of technological coverage; leveraging advanced semantic approaches (e.g., comparative description strength, based on semantic similarity to existing technologies) to optimize differentiation indicators; and finally, applying methods like conditional random fields (CRF) to identify applicant entities and constructing dynamic knowledge graphs of corporate technology portfolios, thereby achieving a more comprehensive evaluation of an enterprise’s innovation capabilities and market impact.

### The implications of the findings for technology innovation management

The AI-driven patent valuation approach exemplified by the BioTexVal model in this study can contribute to the advancement of technological innovation management by enabling more accurate and data-centric decision-making. With an 88.38% accuracy rate in predicting patent value for biomedical textiles, BioTexVal integrates BERT-based textual analysis and structured metrics (e.g., patent families, citations, transfer frequency) to identify high-potential patents early in their lifecycle. Such predictive capabilities assist research institutions, investors, and policymakers in optimizing resource allocation by focusing on R&D projects with stronger commercial and technological prospects while also informing strategies on which patents to retain, license, or further develop. Additionally, the model’s emphasis on global indicators—such as robust international patent filing—illustrates the importance of market expansion and cross-border collaborations in maintaining competitive advantages. By automating patent evaluation, organizations can reduce reliance on subjective expert judgment, streamline portfolio management, and refine IP strategies to better align with long-term innovation goals. Policymakers and patent offices may also find value in integrating BioTexVal-like tools into examination workflows, potentially improving the identification of high-impact patents and tailoring incentives to support emerging innovation clusters. Collectively, these insights suggest that advanced machine learning models can play a valuable role in supporting strategic innovation management, both in biomedical textiles and across other technology-intensive sectors.

As mentioned in the theoretical background, this study does not differentiate among various concepts of patent value—such as gross social value, private value, net social value, or purely technological value—when using forward citation counts as a proxy for patent value. The approach employed here thus aligns with much of the existing literature, treating patent value as a broad, unified concept tied to citation frequency. However, it should be noted that such a proxy does not fully disentangle the complexities inherent in different notions of value. Although forward citations typically indicate a patent’s technological or academic impact and often show a strong positive correlation with its economic returns, they do not reflect actual economic value.

## Conclusion

This study introduces BioTexVal, a machine learning model for patent value grading in the biomedical textiles domain. BioTexVal integrates textual embeddings generated by a pre-trained language model (BERT) with structured patent indicators (such as patent family size and forward citation counts) through a stacking ensemble approach that employs base learners (e.g., Random Forest, XGBoost, LightGBM) and a meta-model (e.g., logistic regression). Experimental results indicate that BioTexVal achieves an accuracy of 88.38% in classifying the value grades of biomedical textile patents, offering a practical analytical tool to identify high-value patents, optimize resource allocation, and guide strategic decisions.

Despite the broad adoption of average annual forward citations as a patent value proxy, this metric alone can be limited. The association between forward citations and economic value is not strictly linear. To more accurately capture a patent’s real-world value, it can be beneficial to integrate industry-specific public or proprietary data into traditional patent databases. Additionally, incorporating first-citation speed and key commercialization milestones (e.g., initial authorization, early-stage funding) with temporal or sequential features can further enhance the evaluation of patent value.

In addition to refining valuation indicators, the stacking technique employed by BioTexVal significantly improves predictive accuracy but also increases computational demands. Training multiple base models and a subsequent meta-model can be time-intensive and resource-heavy, especially for large-scale patent datasets. As the number of models or the depth of stacking grows, computational costs can rise exponentially, potentially limiting broader applicability. To address these constraints, advanced optimization methods—such as knowledge distillation, incremental learning, or lightweight ensemble architectures—could help maintain robust performance while minimizing training overhead.

Although BioTexVal shows promising results in biomedical textiles, cross-domain validation is necessary for generalization. Different industries can exhibit distinct patenting norms, citation behaviors, and innovation cycles, requiring careful tuning and additional training data to confirm the model’s applicability beyond biomedical textiles. Addressing these issues involves integrating complementary valuation metrics (like licensing revenue, commercialization success, and first-citation speed), exploring efficient model architectures that can handle large datasets, and testing the methodology on patent corpora from various technological sectors.

Future research endeavors can further refine BioTexVal and advance the broader field of patent value assessment. First, large language models (LLMs)—including GPT-4 [84], DeepSeek V3 [85], LLaMA 3.0 [86], or Claude-3.5—could be augmented with domain-specific modules (e.g., vision encoders for patent diagrams) to deepen contextual reasoning and improve prediction. Second, parameter-efficient fine-tuning techniques (e.g., LoRA) [87] might adapt these LLMs to domain-specific patent data with moderate computational needs. Third, knowledge distillation from larger LLMs (e.g., GPT-4) could generate specialized but smaller models suitable for resource-constrained environments [88]. Finally, reinforcement learning (RL) may help dynamically optimize valuation criteria by balancing traditional citation-based metrics with key economic indicators. Retrieval-augmented generation (RAG) approaches could integrate external data—such as licensing trends or patent assignee histories—to mitigate citation biases [89]. These enhancements may further capture nuanced innovation signals, enrich economic context, and advance the accuracy and utility of patent assessments across diverse domains.

## Supporting information

S1 Table List of features with the ID. (DOCX)

S2 Table Example of patent text information. (DOCX)
