# Supplementary material for: Patent value prediction in biomedical textiles: A method based on a fusion of machine learning models
Source: PLoS One. 2025 Apr 24;20(4):e0322182. doi: 10.1371/journal.pone.0322182 (PMC12021132; doi:10.1371/journal.pone.0322182)
Supplement: S1 Table — (DOCX) [file pone.0322182.s001.docx]

**S1 Table. list of features with the ID.**

| **ID** | **Structured Patent Indicators** | **ID** | **Structured Patent Indicators** | **ID** | **Structured Patent Indicators** |
| --- | --- | --- | --- | --- | --- |
| 1 | IPC Classification Count | 13 | Number of Family Citations | 25 | IPC Codes |
| 2 | Number of Derwent Manual Codes | 14 | Family Patents Counts | 26 | Derwent Manual Codes |
| 3 | Derwent Classification Count | 15 | Transfer Count | 27 | Patent Type |
| 4 | Year | 16 | Number of Pledges | 28 | Language |
| 5 | Month | 17 | Number of Proceedings | 29 | Publication Country |
| 6 | Date | 18 | Shared Value | 30 | Inventor's Country/Region |
| 7 | Pages | 19 | Technical Stability | 31 | Legal Status |
| 8 | Claims Count | 20 | Technical Advancement | 32 | Legal Event |
| 9 | Independent Claims Count | 21 | Scope of patent protection | 33 | Title |
| 10 | Dependent Claims Count | 22 | Cited Patent Documents Count | 34 | Abstract |
| 11 | Number of Words in the First Claim | 23 | Cited Scientific Publications Count | 35 | Average annual forward citations |
| 12 | Number of Inventors | 24 | Derwent Class Codes |  |  |

A list used to identify the feature**.**
