# Supplementary material for: Patent value prediction in biomedical textiles: A method based on a fusion of machine learning models
Source: PLoS One. 2025 Apr 24;20(4):e0322182. doi: 10.1371/journal.pone.0322182 (PMC12021132; doi:10.1371/journal.pone.0322182)
Supplement: S2 Table — (DOCX) [file pone.0322182.s002.docx]

**S2 Table. Example of patent text information.**

| **ID** | **Title** | **Abstract** |
| --- | --- | --- |
| 1 | Making a unitary fibrous structure for use in e.g. air, water, and ... | Making a unitary fibrous structure includes providing a fibrous web ... |
| 2 | Preparation of a fibrous structure useful filters for e.g. air, oil and ... | Preparation (P1) of a fibrous structure involves providing a mixture (m1) of synthetic fibers ... |
| 3 | Medical dressing useful in healing of wounds has antimicrobial ... | A medical dressing has a layered fabric comprising a hydrophilic inner layer ... |
| 4 | Absorbent core for absorbent article, e.g. diaper, has ... | An absorbent core for an absorbent article, has an absorbent material ... |
| 5 | Absorbent core for absorbent article, has second surface ... | An absorbent core for an absorbent article, has a discontinuous layer of absorbent ... |
| 7 | Tissue implant for use in repair of soft tissue injuries, includes biocompatible ... | A tissue implant comprises a biocompatible polymeric foam and a reinforcement member ... |
